# Effects of Water-Based and Underwater Assistance Methods on the Hole Quality of Silicon Nitride Ceramics Using a Picosecond Laser

**DOI:** 10.3390/mi16060651

**Published:** 2025-05-29

**Authors:** Jie Zhang, Liang Wang, Yongchao Shi, Song Yao, Kaibo Xia

**Affiliations:** 1 School of Mechanical Engineering, Jiangsu University, Zhenjiang 212013, China; 2 Faculty of Mechanical and Materials Engineering, Huaiyin Institute of Technology, Huaian 223000, China

**Keywords:** picosecond laser, water assistance, silicon nitride ceramics, laser power, laser drilling

## Abstract

This study investigated the effects of water-based and underwater assistance methods on the quality of picosecond laser-drilled microholes in silicon nitride ceramics, analyzing the influence of laser power variations in air and aqueous environments on entrance/exit diameters, taper angles, internal wall morphology, surface roughness, and oxygen content. Water-based assistance involved submerging the workpiece’s lower surface while keeping the upper surface in the air, whereas underwater processing involved fully immersing the specimen. The experimental results demonstrated that both aqueous environments effectively improved microhole quality compared to air processing. The water-assisted methods significantly enhanced the entrance/exit morphology by reducing ablation traces and slag deposits. The aqueous medium increased the entrance/exit diameters while decreasing the taper angles and effectively removing debris, thereby reducing internal wall roughness. Underwater processing achieved lower roughness at the hole entrances and middle sections compared to water-based assistance. Both water-assisted methods produced superior internal wall morphology to air processing, with comparable performance. These findings provide valuable references for optimizing water-assisted picosecond laser drilling processes.

## 1. Introduction

Due to their excellent properties, ceramic materials are widely used in new energy, aerospace, microelectronics, and other fields. Among them, silicon nitride ceramics have the characteristics of a low coefficient of thermal expansion, high thermal conductivity, excellent insulation, and high-temperature resistance, and have been applied to the wafer testing of advanced probe card guides and other components [1,2]. Leveraging silicon nitride’s high-temperature resistance (long-term service at 1300 °C) and low-inertia mass characteristics, it is used in aero-engine turbocharger rotors, achieving over a 40% weight reduction compared to metal components while also increasing rotational speed and reducing energy loss. Silicon nitride exhibits a hardness of 16–18 GPa and a flexural strength of 800–1000 MPa, far exceeding traditional probe card substrate materials. Additionally, due to its low dielectric constant, it prevents signal crosstalk and energy loss in high-frequency testing scenarios. Due to the hardness and brittleness of ceramics, traditional mechanical perforation methods struggle to create microholes, making laser processing an effective alternative.

Laser drilling has the advantages of high precision, high efficiency, and no mechanical stress, and has become a key method for manufacturing ceramic microholes. However, this material removal process produces significant thermal effects, resulting in defects such as delamination cracks and recast layers [3]. Compared to conventional laser processing, picosecond lasers minimize the thermal effects on the material. Picosecond laser drilling is a new technology that addresses the problems of large thermal effects, recasting layers, and micro-cracks that exist in long-pulse laser drilling [4,5]. Ackerl [6] compared the ablation behavior of alumina (Al_2_O_3_) and alumina-toughened zirconia (ATZ) ceramics under ultrashort-pulse laser irradiation. A sub-picosecond green (515 nm) laser enabled high-efficiency, low-thermal damage machining; however, in microhole machining, excessive energy density (0.8 J/cm^2^) induced HAZ and Al_2_O_3_ dissolution. Amsellem et al. [7] conducted cutting experiments on SiC using a picosecond laser with a pulse width of 3 ps. Through integrating wobble patterns, they systematically optimized the processing parameters, including focal position, linear speed, and wobble frequency, effectively addressing the re-deposition and thermal damage issues in SiC ceramic machining. Marimuthu et al. [8] conducted deep machining of tungsten carbide using a 300 W high-power picosecond laser. Under optimal conditions (e.g., energy density of 6 J/cm^2^ and pulse duration of 0.9 ps), the material removal rate (MRR) reached 45 mm^3^/min. The shorter pulse (0.9 ps) increased the removal rate by 75% compared to longer pulses (10 ps), while the edge taper was minimized to approximately 15°.

To process a hole with a large depth-to-diameter ratio and maintain processing efficiency, a high laser energy density is required. However, this can lead to heat accumulation, especially at high pulse repetition frequencies. As a result, thermal damage may occur, slag particles can remain on the inner wall of the hole, and micro-cracks and recast layers may also form [9,10]. Therefore, improving the quality of the inner wall of the laser hole and reducing the thermal damage under high laser energy densities and pulse repetition rates have become urgent problems requiring solutions. In recent years, auxiliary technologies such as ultrasonic vibration [11], magnetic field assistance [12], and water assistance [13] have been increasingly integrated into laser drilling processes. As an advanced manufacturing process, water-assisted laser processing can effectively reduce or eliminate the above defects. Common water-assisted laser drilling processes include underwater-assisted laser drilling [14,15], water-jet-assisted laser drilling [16], and water-jet-guided laser drilling [17,18]. Garcia-Giron et al. [19] found that the ablation yield of water-assisted laser processing was higher than that obtained when the sample was treated in air, and the ablation yield of glass–ceramics, 8YSZ, and alumina was 3.88, 1.76, and 26.06 times higher, respectively. This improvement was due to the mechanical enhancement created by the high pressure and cavitation bubbles that appeared in the liquid during the ablation process. Zhao et al. [20] studied the influence mechanism of water layer thickness on processing quality in water-assisted laser processing. Fluent software was used to simulate the flow field characteristics for different water layer thicknesses (1, 2, 3, and 5 mm), and experimental verification was conducted. It was found that under a 1 mm water layer, the flow was laminar (Reynolds number ≈ 1200) with a uniform and stable velocity distribution, while thicker water layers resulted in turbulent flow (Reynolds number ≥ 2400), which caused violent fluctuations, laser refraction and scattering, and a reduction in processing accuracy. Zhang [21] proposed a backwater-assisted flow water laser drilling method. By introducing flowing water to remove the bubbles and debris from the back of the workpiece, the clogging issues in traditional backwater-assisted laser drilling during microhole machining with a high depth-to-diameter ratio were solved. Feng et al. [22] used underwater-assisted picosecond laser drilling to drill holes in zirconia and systematically studied the influence of process parameters on the geometrical quality of the holes. The results showed that drilling in a water environment had little effect on the mechanical properties of the material surrounding the drilling holes, and it helped to reduce micro-cracks and internal surface roughness.

In summary, to further improve the quality of drilling, domestic and foreign scholars have proposed a water-assisted laser drilling method. Their results have shown that water-assisted laser drilling can reduce taper, thermal effects, micro-cracks, residual stress, and recast layers. To further optimize the water-assisted picosecond laser drilling method and better understand the differences between various water-assisted methods, this study developed a water-assisted picosecond laser hole-making platform and investigated the influence of different laser powers on the geometric structure and inner wall quality of the holes. Experiments on picosecond laser drilling of silicon nitride using two different water-assisted methods, water-assisted and underwater-assisted, were carried out. The results were compared to changes in hole quality under air conditions, and the influence mechanism was analyzed, which provided a theoretical basis for picosecond laser hole-making.

## 2. Experimental Setup and Procedure

**Figure 1:**
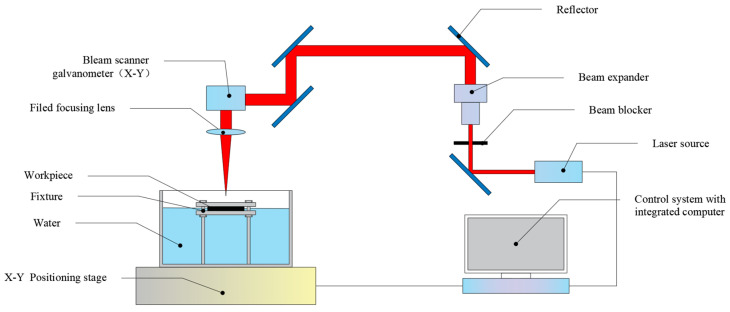
Schematic diagram of the picosecond laser processing system.

Figure 1 shows the picosecond laser machining center, composed of a mechanical motion system, a water-assisted fixture system, an optical path control system, and a picosecond laser source, which can realize concentric circle scanning path processing. The laser equipment included a laser processing center produced by Beijing Laize Photonics Co., Ltd. (Beijing, China), equipped with an FSLAB-PICO series picosecond laser from Beijing Laize Photonics Co., Ltd. (Beijing, China). This laser is mainly used in the field of precision and fine machining, is suitable for processing various materials, and has more advantages for brittle ceramic materials. The picosecond laser generated pulsed light with a laser frequency of 200 kHz, a wavelength of 355 nm, and a pulse width of 10 ps. The laser power was controlled by adjusting the single-pulse energy. The laser beam passed through a beam expander system (input beam diameter: 3 mm; expansion ratio: 4) for collimation and diameter enlargement, reducing the divergence angle to ensure uniformity of the incident spot on subsequent focusing lenses and improve the consistency of far-field energy distribution. The expanded beam was then directed via mirrors to a galvanometer scanner system that achieved high-speed two-dimensional planar scanning through rapid deflection control. Finally, the laser beam was focused onto the workpiece surface through a focusing lens. Table 1 shows the parameters of the picosecond laser system. The experiments mainly compared the conditions of laser drilling in air, water-based, and underwater environments.

The experiment primarily consisted of three parts: sample preparation, laser drilling, and measurement characterization. First, silicon nitride ceramic plates (specifications: 100 × 100 × 1 mm^3^) were cut into specimens (specifications: 20 × 10 × 1 mm^3^) using a millisecond laser. The laser parameters included a wavelength of 1064 nm, a pulse width of 1 ms, a repetition frequency of 60 Hz, a single-pulse energy of 2 J, and a laser feed rate of 40 mm/min. The chemical composition of the silicon nitride ceramic is listed in Table 2. After cutting, the specimens were placed in a beaker containing anhydrous ethanol. The beaker with the specimens and ethanol cleaning solution was then placed in an ultrasonic cleaner to ensure the specimens’ surfaces were thoroughly cleaned, avoiding any contamination that might have affected the experiment.

**Figure 2:**
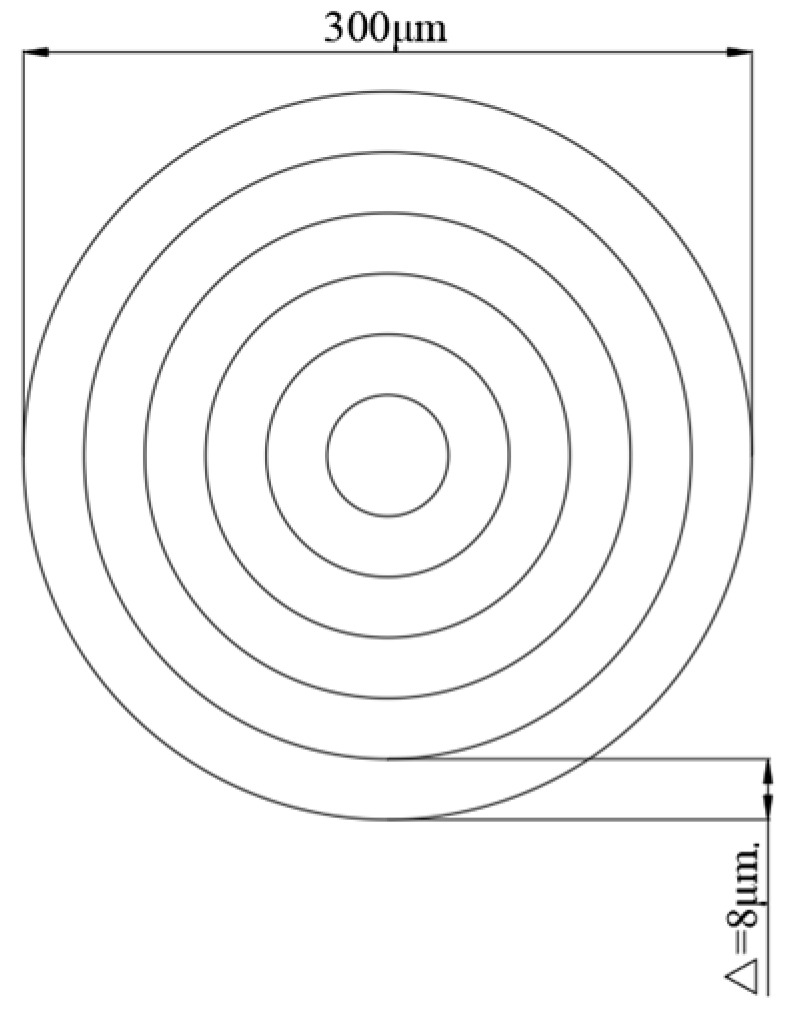
Picosecond laser processing scanning path diagram.

**Figure 3:**
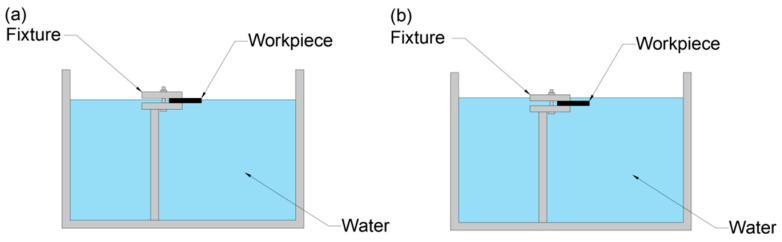
Schematic diagram of the water-assisted picosecond laser processing: (**a**) water-based assistance; (**b**) underwater assistance.

The second part involved laser drilling. The preset aperture of this experiment was 300 μm, and the experimental material was a silicon nitride ceramic sheet with a thickness of 1 mm. As shown in Figure 2, the experimental path was concentric circles with a maximum circle diameter of 300 μm and an interval of 8 μm between each circle. The geometric structure and the difference in the inner wall quality of the holes made with air, water-based, and underwater assistance were studied experimentally by changing the laser power. The laser parameters in the experiment are shown in Table 3. First, drilling was performed under atmospheric conditions, with each hole using identical laser parameters repeated three times. Then, the water medium was sequentially injected at different liquid level heights into the fixture, repeating the same parameters as atmospheric drilling to conduct water-based and underwater-assisted laser drilling operations. As shown in Figure 3, the water level of the water-based condition reached the middle of the workpiece, approximately 0.3 mm away from the upper surface of the workpiece, while the water level of the underwater condition was approximately 0.7 mm away from the upper surface of the workpiece [23]. To ensure that the water levels met the design requirements, the water requirement was increased after calculation (the water medium was ordinary pure water, and the water quantity was measured using a measuring cylinder).

After drilling completion, the processed specimens required various measurement characterizations. Ultrasonic cleaning was employed to remove residual impurities before capturing the entrance/exit morphologies of the holes. Subsequently, diamond grinding discs (specifications: 400 and 800 mesh) were used to polish the entrance/exit areas to remove molten slag for hole diameter measurement. Prior to cross-sectional imaging, the specimens’ sides were preprocessed using a metallographic grinding machine with retained machining allowance. Diamond grinding discs were then manually applied for precision grinding to avoid excessive material removal and ensure measurement accuracy. Post-grinding, the specimens underwent polishing using a polishing machine, followed by additional ultrasonic cleaning to prevent impurities from blocking the microholes or scratching the inner walls. The cross-sectional morphology and inner wall surface characteristics of the holes were observed using a confocal laser scanning microscope (CLSM), with quantitative characterization and measurement of the inner wall surface roughness performed.

## 3. Experimental Results and Discussion

### 3.1. Effect of Laser Power on the Hole Entrance and Exit Morphologies

**Figure 4:**
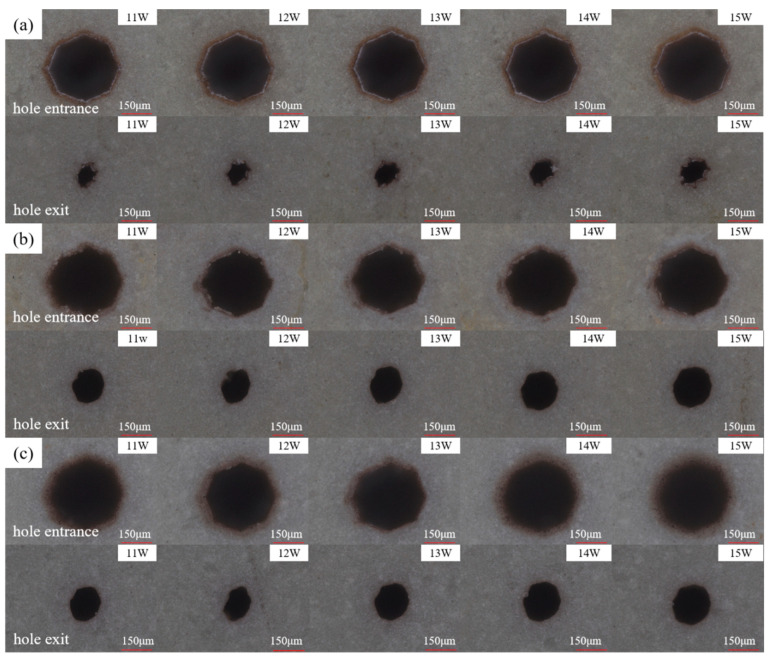
Effect of laser power variation on the entrance and exit morphologies of picosecond laser drilling: (**a**) in air, (**b**) water-based assistance, and (**c**) underwater assistance.

Figure 4 shows the entrance and exit morphologies of picosecond laser drilling in air, water-based, and underwater environments. Figure 4a shows the entrance and exit morphologies of picosecond laser drilling in an air environment. It can be observed that the entrance aperture is significantly larger than the exit aperture. This phenomenon is due to the interaction between the laser and the material during the picosecond laser hole-making process, which generates plasma at the entrance. Inside the hole, the plasma mixes with molten matter, water vapor, etc., to form a plasma plume [24]. These physical phenomena have a shielding effect on laser energy and hinder the effective absorption of laser energy by materials. With the increase in hole-making depth, the material removal rate decreases gradually. In addition, during laser processing, the *Z*-axis position remains constant, and as the depth increases, the relative focal length increases, resulting in a decrease in laser energy utilization. These two factors work together, resulting in the appearance and size of the exit being smaller than that of the entrance. The polygonal features observed in the entrance/exit morphologies are primarily attributed to the lack of arc interpolation mode in the control software, which approximates circular machining using linear segments, combined with the asymmetry between P- and S-polarized light in the laser beam that causes non-uniform material absorption of the laser energy [25]. Near the entrance and exit of the holes is a ring of ablative marks, accompanied by a small amount of redeposited material residue and molten material, which was caused by thermal effects under the high energy density and high repetition frequency conditions.

Figure 4b shows the entrance and exit morphologies of picosecond laser drilling in a water-based environment. The ablation marks at the entrance of the microholes are significantly reduced, as are the redeposited material residues and molten matter. The ablation marks at the exit of the microholes are almost gone, and the redeposited material residues and melts are also nearly absent. This is because, during the process of water-based picosecond laser hole-making, the water medium plays a cooling role, which greatly inhibits the thermal effect generated during picosecond laser drilling. After the through hole is formed, the water medium enters the microhole from the exit and interacts with the laser. The water absorbs the laser’s energy, producing bubbles and water vapor. The bubbles then burst, and the floating process produces an impact force, causing water to flow into the hole and effectively carry away the redeposited material residue and melt. Therefore, under water-based conditions, the redeposited material residues and melts around the microhole entrance and exit almost disappeared, the ablation marks were reduced, and the surface was cleaner.

Figure 4c shows the entrance and exit morphologies of underwater picosecond laser drilling. Compared to the results of hole-making in air and water-based environments, the ablation marks at the entrance of microholes have almost disappeared; only some traces can be observed at high power (14 and 15 W), and the redeposited material residues and melts have basically disappeared. There are almost no ablative marks or melt residue at the exit of the microholes, which is consistent with the phenomenon in the water-based environment. This is due to the cooling effect of the water medium in the process of underwater picosecond laser hole-making, which effectively reduces the thermal effect produced by picosecond laser drilling [26,27]. Different from the water-based environment, the water medium can interact with the laser beam in the initial stage of underwater laser hole-making. Before the microhole is fully opened, the water medium can enter the microhole from the entrance, producing water vapor and bubbles under the action of the laser. The impact force generated by bubble rupture and floating, as well as the flow of water in the hole, takes away the redeposited material residue and melt from the entrance. After the hole is fully opened, the water medium passes through the entire inside of the microhole, producing bubbles and water vapor. The impact force generated by bubble rupture and upward motion, as well as the flow of water in the hole, can then carry away the redeposited material residue and melt from the entrance and exit at the same time. Therefore, under underwater conditions, the thermal effect at the entrance was smaller than that under water-based conditions, and the ablation marks were less obvious, while the situation at the exit was basically the same. There was also a dark brown area at the entrance of the underwater specimen. In general, during picosecond laser processing under water-based conditions, the workpiece was not fully immersed in the water, which prevented the water from filling the inside of the microholes. Consequently, it was difficult to achieve effective removal of debris and melt during underwater-assisted laser processing of microholes. This resulted in the entrance morphology under water-based conditions being less clean than that under underwater conditions. At the same time, at the entrance under underwater conditions, the laser-induced plasma generated a shock wave [28], which acted on the workpiece surface. The water vapor and water medium contained plasma, melt, and material debris, which intensified the heat exchange on the workpiece surface, resulting in the formation of a mixing area around the hole entrance containing a small amount of material debris, but no melt precipitation phenomenon.

### 3.2. Influence of Laser Power on Hole Diameter

**Figure 5:**
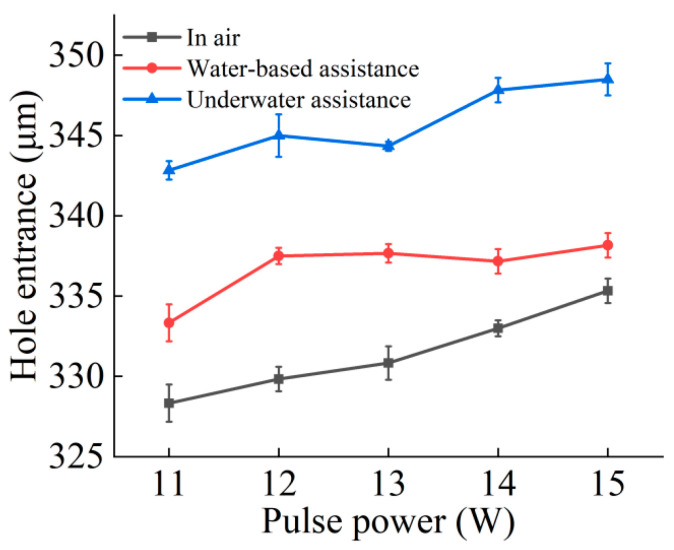
Effect of laser power variation on the hole entrance diameter of picosecond laser hole-making.

**Figure 6:**
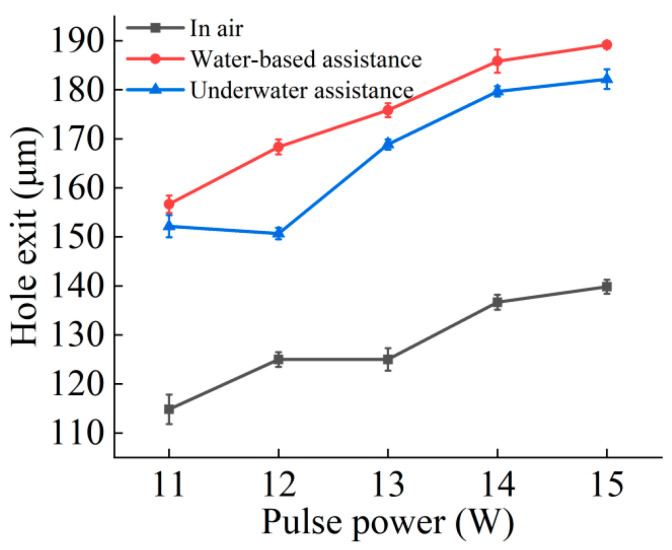
Effect of laser power variation on the hole exit diameter of picosecond laser hole-making.

Figure 5 and Figure 6 respectively show the curve changes of micropore entrance and exit aperture under air, water-based, and underwater conditions with changing picosecond power. In general, under these three conditions, the diameter of the exit and entrance of the microholes increased with the increase in laser power. This is because, in the case of constant repetition frequency, the increase in laser power resulted in an increase in laser energy. As laser energy increased, the amount of energy the material could absorb and use increased, facilitating the removal of the material; therefore, the diameter of the entrance and exit increased with increasing laser power.

Compared to the air environment, the entrance and exit diameters of the microholes increased under the water-based conditions. Once the microhole was complete, water flowed into the inside of the microhole from the exit and interacted with the laser and material. The water absorbed the laser energy, producing bubbles and water vapor. The rupture and floating process of the bubbles produced an impact force, coupled with the water flowing through the hole, thereby weakening the plasma shielding effect on the laser energy and effectively improving the material removal rate. In addition, the laser induced a shock wave in the plasma in the water, which also produced an impact force, further increasing the material removal rate and resulting in an increase in the entrance and exit apertures. At the workpiece entrance, the water medium could not completely cover the whole area, limiting its effect on the material removal rate. However, with increasing laser processing depth, the water medium removed impurities such as material debris from the microhole, reduced the barrier above the processed material, improved the utilization rate of the laser for the material to be processed, and greatly increased the material removal rate at the exit.

In underwater-assisted processing, the entrance diameter was slightly larger than the entrance diameter under water-based conditions, while the exit diameter was slightly smaller. Compared to in air, the diameters of the entrance and exit of the microhole increased under underwater conditions. From the beginning of microhole laser processing, water could enter the hole. The water flow, along with the generation and rupture of bubbles and other phenomena, effectively promoted the removal of materials. At the same time, the water enhanced the shock wave generated by the laser-induced plasma, and the resulting impact force further increased the melt and debris removal. After the workpiece was drilled through, water was able to fully enter the hole, increasing the material’s absorption of laser energy. As a result, the material near the bottom of the hole absorbed more energy, leading to an increase in the exit diameter of the microhole. In addition, some water splashed around the entrance of the hole, which also improved the absorption rate of laser energy. Because the *Z*-axis position remained unchanged when the laser acted on the material workpiece, the distance between the material processing surface and the water surface gradually increased with the increase in hole depth, and the water depth continued to deepen, which exceeded the range where the depth of the water medium had the best effect on the material removal rate. Therefore, the aperture of the underwater exit was larger than that of the air exit, but smaller than that of the water-based exit.

### 3.3. The Effect of Laser Power on the Cross-Sectional Morphology and Taper of Microholes

**Figure 7:**
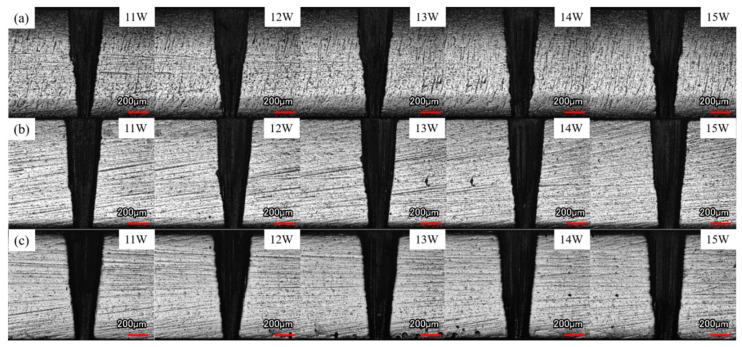
Cross-section morphologies of holes drilled at different pulse powers: (**a**) in air, (**b**) water-based assistance, and (**c**) underwater assistance.

**Figure 8:**
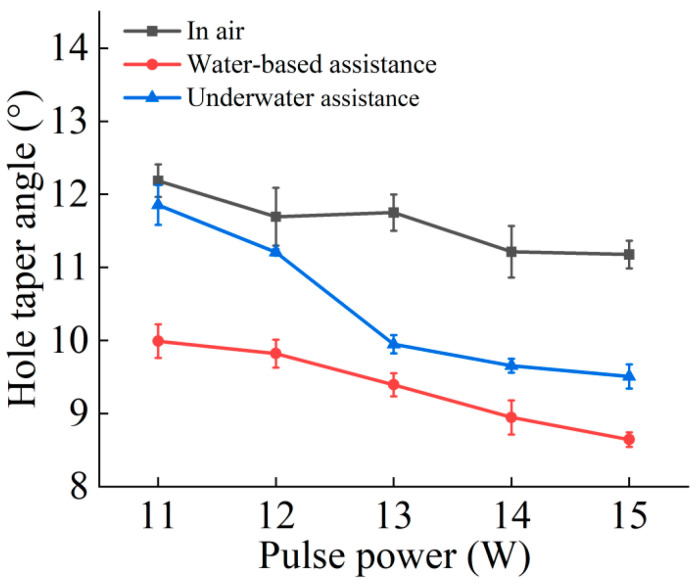
The effect of laser pulse power on hole taper angle: in air, water-based assistance, and underwater assistance.

The cross-sectional morphology and taper of the microholes are illustrated in Figure 7 and Figure 8. A comprehensive analysis of these two figures reveals that under the three drilling conditions—air, water-based, and underwater—the taper of the microholes decreases with increasing picosecond laser power. The reason is that as the laser power increases, the laser energy that the internal material of the microholes can absorb and utilize in the three drilling environments increases, significantly improving the material removal rate at the exit. This is consistent with the phenomenon of the hole diameter increasing with laser power, as mentioned earlier, and exhibits inherent consistency.

Compared to the air environment, the taper of microholes was smaller under water-based and underwater conditions. During laser processing of workpieces in air, plasma above the entrance shielded the incident laser; inside the hole, the plasma plume formed by the interaction of plasma and molten material also had a shielding effect on the laser energy, thereby reducing the material removal rate. Under water-based conditions, on the one hand, after the laser drilled through the workpiece, water quickly rushed into the microhole and evaporated due to heating, effectively suppressing the shielding effect of the plasma plume. On the other hand, the laser entering the microhole caused water to generate microflow and bubbles inside the hole. The impact force generated by the rising and bursting of bubbles significantly improved the material removal rate. In addition, the shock wave induced by the laser in the water also produced a mechanical impact force, further improving the material removal rate. These factors resulted in a significantly higher material removal effect at the exit under water-based conditions compared to air conditions, thereby reducing the taper of the microholes. Under underwater conditions, from the initial stage of laser processing of the microhole, the water medium entered the hole and flowed, generating bubbles and water vapor, which suppressed the shielding effect of the plasma plume and increased the material removal rate of the microhole. After the workpiece was drilled through, the water continued to act inside the microhole, effectively improving the material removal rate at the exit and thereby reducing the taper. Ultimately, the taper of microholes under the two water-assisted conditions was significantly reduced compared to that in the air.

As shown in Figure 8, the taper of the hole under underwater conditions is greater than that under water-based conditions. This is because in the underwater environment, when the thickness of the water layer exceeded a certain threshold, the material removal rate tended to decrease. During the laser drilling process, as the depth of the hole increased, the distance between the material processing surface and the water surface gradually increased, and accordingly, the equivalent water layer thickness increased. This change resulted in a relatively higher material removal rate at the entrance and a lower material removal rate at the exit under underwater-assisted processing conditions, ultimately leading to a greater hole taper under underwater conditions than under water-based conditions.

### 3.4. The Effect of Laser Power on the Inner Wall Morphology of Microholes

**Figure 9:**
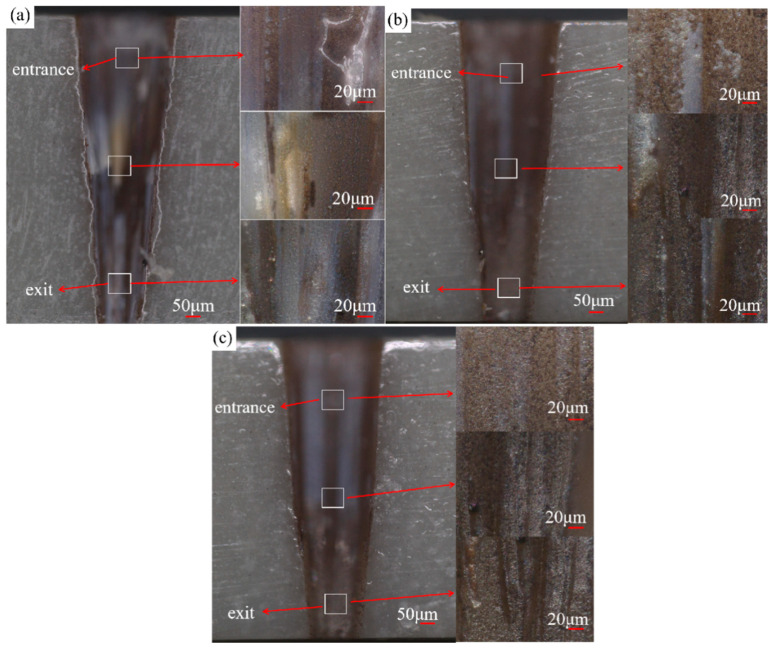
Appearance diagram of the hole sidewall: (**a**) in air, (**b**) water-based assistance, and (**c**) underwater assistance.

Figure 9 presents the two-dimensional morphologies of the entrance, middle, and exit of the inner walls of microholes under air, water-based, and underwater conditions. As shown in Figure 9a, under air conditions, defects such as material removal residues and protrusions appear near the entrance, middle, and exit of the microhole inner walls. This is because in the air environment, material impurities and debris were not promptly removed and thus adhered to the inner walls. The closer to the exit, the more irregular the microhole inner wall becomes, presenting a furrowed appearance. The reason is that under air conditions, as the hole depth increased, the shielding effect of the plasma plume on the laser energy became stronger, resulting in a worse material removal effect on the microhole inner wall. Additionally, the asymmetry of the laser’s P and S light caused uneven absorption of laser energy by the material, which is also one of the main factors. Compared to water-based and underwater conditions, the microhole inner walls under air conditions are partially covered with black and brownish–yellow areas, which were caused by the thermal effects of laser ablation under high energy density and high repetition frequency.

Figure 9b shows the situation under water-based conditions. Compared to air conditions, the microhole inner walls are much smoother, with almost no material removal residues or protrusions. This is because during laser processing, after the microhole was drilled through, the water medium entered the microhole. The shock wave induced by the laser in the water, along with the water flow and the impact force generated by the bubbles, removed debris, molten material, and material removal residues, making the microhole inner walls smooth. At the same time, the laser ablation area disappeared, thanks to the cooling effect of the water medium, which reduced the thermal effects of laser ablation. Compared to air conditions, the microhole inner walls under water-based conditions hardly show any furrowed appearance. This is because, during laser processing, the water medium entered the microhole and effectively suppressed the shielding effect of the plasma plume, enhancing the absorption of laser energy by the microhole inner wall, making it more uniform, and improving the material removal rate of the microhole inner wall. Moreover, the water medium could promptly remove material debris, making the microhole inner walls smooth and flat, with almost no furrowed appearance.

As shown in Figure 9c, the overall inner wall morphology of the microhole under underwater conditions is basically consistent with that under water-based conditions. However, there are differences. The part near the entrance under underwater conditions is slightly cleaner than that under water-based conditions. This is because, under underwater conditions, before the microhole was drilled through, the water medium could enter the microhole and remove debris and molten material. After the microhole was drilled through, the water medium could fully fill the entire microhole, thus thoroughly removing impurities from its entrance, middle, and exit. Under water-based conditions, even after the microhole was drilled through, the water medium could not fully fill the entrance of the microhole, causing some parts of the entrance to occasionally come into contact with air. As a result, some debris and molten material could not be promptly removed as they were under underwater conditions, and thus, they adhered to the inner walls. Therefore, the entrance inner wall under underwater conditions is slightly cleaner than that under water-based conditions.

Overall, under the three conditions, the greater the depth, the more severe the furrowed phenomenon. In addition to the aforementioned reason that laser energy was shielded, this processing method maintained a stationary *z*-axis. As the depth increased, the energy loss intensified, resulting in a lower energy utilization rate. Under the two water-assisted conditions, the water medium significantly improved the laser energy utilization rate, thereby significantly improving the furrowed phenomenon.

### 3.5. The Effect of Laser Power on the Inner Wall Roughness of the Microholes

**Figure 10:**
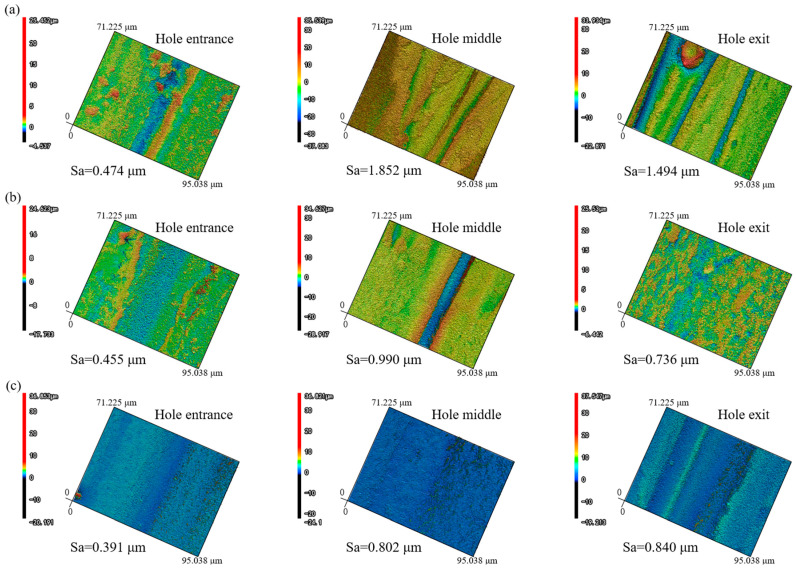
3D sidewall surface morphologies: (**a**) in air, (**b**) water-based assistance, and (**c**) underwater assistance.

**Figure 11:**
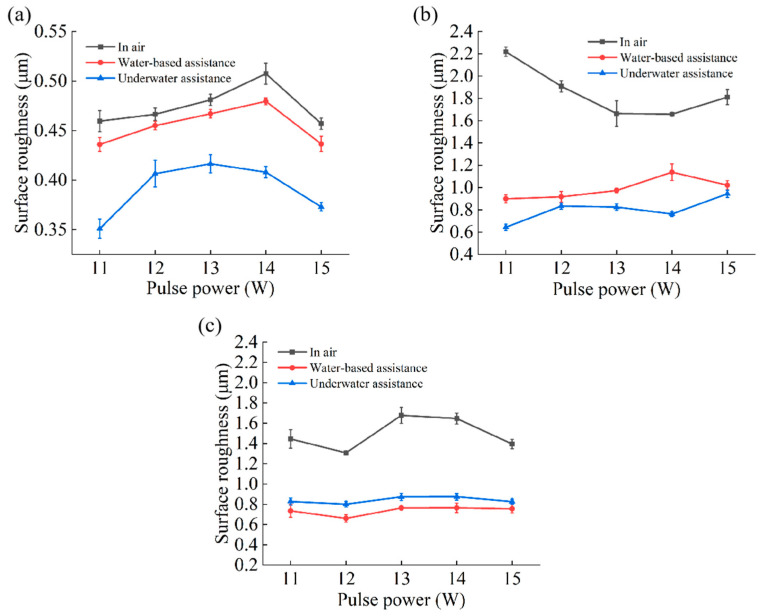
Effect of pulse power on hole sidewall roughness: (**a**) hole entrance, (**b**) hole middle, and (**c**) hole exit.

Figure 10 and Figure 11 present the three-dimensional morphologies and roughness characteristics of the inner walls of the microhole entrance, middle, and exit under air, water-based, and underwater conditions.

Under air conditions, the roughness of the microhole entrance was relatively low, while the roughness of the middle and exit was higher. This is because in the air medium, as the depth of microhole processing increased, the laser energy was gradually shielded by the plasma. The closer to the microhole exit, the lower the utilization rate of laser energy, resulting in an insufficient and non-uniform material removal process, which gave the inner wall a furrowed appearance. Moreover, a large amount of molten material and other impurities adhered to the inner wall surface, further increasing the roughness of the middle and exit of the microhole under air conditions.

Under water-based conditions, after the workpiece was drilled through, the laser continued to process, and the water medium entered the microhole, generating turbulence, bubbles, and water vapor inside the hole. The water flow, the rising of bubbles, and the impact force generated by their bursting can effectively remove debris and molten material, while the evaporation of water vapor can suppress the shielding effect of plasma on the laser [29,30]. Overall, this mechanism significantly enhances the ability to suppress the shielding effect of the plasma plume, thereby effectively increasing the material removal rate. Compared to air conditions, the roughness of the microhole inner wall under water-based conditions was significantly reduced. The material removal rate at the middle and exit of the microhole was greatly increased, and the amount of adhering material was significantly reduced; thus, the reduction in roughness of the middle and exit was more pronounced than that of the entrance.

Under underwater-assisted conditions, there was a significant difference compared to under water-based conditions. Since the workpiece was completely submerged underwater, the water medium could enter the microhole before the laser drilled through the workpiece. At this time, the water medium not only played a cooling role to reduce thermal effects but also generated bubbles and water vapor, thereby effectively increasing the material removal rate. Once the workpiece was drilled through, the water medium continued to surge inside the microhole, generating bubbles that effectively removed debris and molten material. This resulted in a significant reduction in the inner wall roughness of the microhole under underwater-assisted conditions compared to air conditions. In the middle and exit regions, the material removal rate increased, the furrowed phenomenon was reduced, and the amount of adhering molten material and other impurities also decreased. Therefore, the reduction in roughness of the middle and exit was more pronounced.

Compared to water-based assistance, underwater assistance was more effective in suppressing the shielding effect of the plasma plume, especially near the entrance. This is because, under water-based conditions, the entrance was exposed to the air environment, and the water medium could not fill the entrance area as it could under underwater conditions. This, to some extent, affected the material removal rate and made it easier for molten material and other impurities to adhere to the inner walls near the entrance. Since the middle part was connected to the entrance, it was also affected to some extent. Therefore, the roughness of the entrance and middle inner walls under underwater conditions was slightly lower than that under water-based conditions. However, as the depth increased, the material removal rate of the underwater exit decreased and the removal process became relatively non-uniform, resulting in a slightly higher roughness of the underwater exit than of the water-based exit.

Overall, under both water-based and underwater-assisted conditions, the roughness at the entrance was the lowest, while the roughness at the middle and exit was relatively higher. This is because, as the processing depth increased, the relative thickness of the water medium increased, leading to a decrease in the utilization rate of laser energy. This, in turn, caused insufficient and non-uniform material removal from the inner walls of the middle and exit regions, resulting in a furrowed appearance. Consequently, the roughness of the middle and exit regions under water-based and underwater conditions was greater than that of the entrance.

## 4. Conclusions

This study experimentally investigated the effects of water-assisted and underwater picosecond laser drilling methods on the geometric morphology and hole wall quality of microholes under different laser powers, providing a new process for water-assisted picosecond laser drilling. The following conclusions can be drawn:

1. Compared to air processing, the entrance and exit morphologies of holes drilled under static water-based and static underwater conditions were significantly improved. The ablation marks were smaller, and there was less slag and other adhering materials, resulting in cleaner entrances and exits. This was due to the cooling and flushing effects of the water medium, which suppressed thermal effects and removed impurities.
2. Compared to air processing, the diameters of the entrance and exit of the microholes drilled under water-based and underwater conditions were both larger, with a more noticeable increase in the exit diameter. The entrance diameter under underwater conditions was larger than that under water-based conditions, while the exit diameter was smaller. This difference arises from the different ways and extents of interaction between the water medium, laser, and material in different water-assisted environments. Under underwater conditions, the water medium was fully involved from the beginning of the processing, while under water-based conditions, the way the water medium intervened after the workpiece was drilled through differed from that under underwater conditions, resulting in different characteristics of the exit diameter.
3. Compared to air processing, the taper of the microholes drilled under water-based and underwater conditions was reduced. The water medium suppressed the shielding effect of the plasma plume on the laser energy, resulting in more uniform hole wall processing. Due to the difference in the diameters of the entrance and exit holes, the taper of those drilled under underwater conditions was slightly larger than that of those under water-based conditions.
4. Both water-assisted conditions result in fewer adhering materials on the inner walls of the microholes, making them smoother. However, there were still furrow-like features near the exit. Overall, the roughness of the inner walls was reduced under the water-assisted conditions, with a greater reduction in the middle and exit regions. Between the two water-assisted conditions, the roughness of the entrance and middle regions under underwater conditions was lower, while the roughness at the exit was essentially the same.

## Figures and Tables

**Table 1:** Parameters of the picosecond laser.

**Wavelength/nm**	**Maximum Power/W**	**Laser Pulse Duration/ps**	**Pulse Repetition Rate/kHz**
355	16	10	200
**Maximum Single Laser Pulse Energy/μJ**	**Spot Roundness**	**Energy Stability/rms**	**Energy Stability/M^2^**
200	>85%	≤1%	<1.3
**Focal Length/** **mm**	**Numerical Aperture (NA)**	**Beam Expansion Ratio**	**Laser Incident Diameter** **/mm**
80	0.1	4	3 ± 0.5

**Table 2:** Chemical composition of the silicon nitride ceramic Si_3_N_4._

**Composition**	**Si**	**N**	**Si** **(** **Separated** **)**	**O**	**Mg**	**Fe**
Mass fraction/%	≤0.08	17.0–21.0	50.0–55.0	≤1.0	2.80–3.30	0.30–0.70
**Composition**	**Mn**	**Ti**	**Phase Content**	**Phase α**	**Particle Size**	**D50**
Mass fraction/%	<0.075	<0.025	Mass fraction/%	>92	μm	≤0.5

**Table 3:** Specific parameters used in the experiment.

**Wavelength (nm)**	**Pulse Power (W)**	**Pulse Width (ps)**	**Spot Diameter (μm)**
355	11/12/13/14/15	10	15
**Repetition Rate (kHz)**	**Scanning Speed (mm/s)**	**Number of Turns (n)**	
200	30	20	

## Data Availability

The original contributions presented in this study are included in the article; further inquiries can be directed to the corresponding author.
